# In memoriam: John Lisman – commentaries on CaMKII as a memory molecule

**DOI:** 10.1186/s13041-018-0419-y

**Published:** 2018-12-28

**Authors:** Mark F. Bear, Sam F. Cooke, Karl Peter Giese, Bong-Kiun Kaang, Mary B. Kennedy, Ji-il Kim, Richard G. M. Morris, Pojeong Park

**Affiliations:** 1 Picower Institute for Learning and Memory, Department of Brain and Cognitive Sciences, Massachusetts Institute of Technology, Cambridge, MA 02139 USA; 2 King’s College London, Department of Basic and Clinical Neuroscience, Institute of Psychiatry, Psychology and Neuroscience, De Crespigny Park, London, SE5 8AF UK; 3 Department of Biological Sciences, Seoul National University, Gwanak-gu, Seoul, Republic of Korea; 4 The Division of Biology and Biological Engineering, California Institute of Technology, Pasadena, CA 91125 USA; 5 Laboratory for Cognitive Neuroscience, Centre for Discovery Brain Sciences, Edinburgh Neuroscience, Edinburgh, EH8 9JZ UK

## Abstract

Shortly before he died in October 2017, John Lisman submitted an invited review to Molecular Brain on ‘Criteria for identifying the molecular basis of the engram (CaMKII, PKMζ)’. John had no opportunity to read the referees’ comments, and as a mark of the regard in which he was held by the neuroscience community the Editors decided to publish his review as submitted. This obituary takes the form of a series of commentaries on Lisman’s review. At the same time we are publishing as a separate article a longer response by Todd Sacktor and André Fenton entitled ‘What does LTP tell us about the roles of CaMKII and PKMζ in memory?’ which presents the case for a rival memory molecule, PKMζ.

**John Lisman 1944–2017**

*Contributed by Karl Peter Giese*

Last October John Lisman sadly passed at the age of 73. John was an exceptional neuroscientist who made a wide variety of seminal contributions. His very highly cited work includes the development of novel theories about the role of bursts in information processing [1], the storage of short-term memories in oscillatory cycles [2], the function of the hippocampal-VTA in long-term memory formation [3], and the CaMKII (Ca^2+^/calmodulin-dependent protein kinase II) hypothesis for memory storage. The latter CaMKII hypothesis was John’s most important contribution. It originated from a theoretical paper in 1985 where he proposed that the molecular basis for memory storage could be an autophosphorylating kinase that persistently maintains activity at synapses [4]. Shortly after John’s publication CaMKII was biochemically purified from synapses and shown to have an autophosphorylation switch. Together, with John’s theoretical paper this led to the CaMKII hypothesis for memory storage [5]. The attractiveness of this hypothesis motivated many eminent neuroscientists to study the function of CaMKII and its isoforms in learning and memory. Accumulating experimental evidences not only supported, but also provided some problems for the CaMKII hypothesis, which motivated refinements that to keep the hypothesis viable [6–9]. Last year John was very delighted to publish experimental proof for his CaMKII hypothesis after more than 30 years of research [9, 10]. Next to his pioneering work on memory storage, John was also a true scholar who was open for discussion of other viewpoints. I experienced this myself when questioning the CaMKII hypothesis over many years. John will be remembered not only for his seminal contributions, but also for the breadth and generosity of his academic spirit in advancing our understanding of the brain.

**The enigma of memory maintenance: Commentary on Criteria for identifying the molecular basis of the engram (CaMKII, PKM ζ)**

*Contributed by Sam F. Cooke and Mark F. Bear*

The late John Lisman was one of the great thinkers of neuroscience. He posed some of the biggest questions and provided eminently testable hypotheses. Amongst his many contributions, he is probably best known for illustrating how the enzyme Calcium/calmodulin-dependent protein kinase II (CaMKII) may serve as a molecular switch to maintain changes in synaptic strength and long-term memory [4, 5, 11, 12].

In the early 1980’s, variations on Hebbian synaptic plasticity had started to predominate as the favoured theoretical mechanism by which learning occurs in the brain, several decades after the key concepts had first been formalized by Donald Hebb [13] and a decade after the first example of lasting Hebbian plasticity, known as long-term potentiation (LTP), was observed in the brain of living organisms [14, 15]. LTP has several remarkable properties: It is “input specific”, meaning that it can occur selectively at a very small fraction of the synapses formed on a given neuron [16]; it is rapidly induced within seconds [15]; and it can last months in vivo [17] and many hours in brain slices [18]. These properties are ideal for encoding episodic memories, but present challenges for understanding the underlying cellular biology. Longevity of LTP could be explained by stable activation of gene expression and new protein synthesis, but how would these new proteins be continuously supplied to (or captured by) only the potentiated synapses, and how would this mechanism account for the rapid induction of LTP at synapses far away from the nucleus? On the other hand, rapid induction and synapse specificity could be readily accounted for by local post-translational modification of pre-existing synaptic proteins, but how would this be maintained in the face of protein turnover?

Much thought has been expended on these questions and the most influential solutions came from Lisman [4] and, independently, from another deep thinker, Francis Crick [19]. To address these twin problems, the concept of a ‘memorase’ was introduced, which is an enzyme composed of multiple subunits that can auto-regulate each other to sustain activity and maintain synaptic change by continually modifying key synaptic proteins. Thus, newly synthesized subunits could be incorporated at any time to replace outgoing subunits and switched into an active form through post-translational modification by a neighbouring subunit, thereby maintaining the overall ‘active’ state of the molecular complex. Lisman and Goldring went so far as to posit the likeliest candidate, CaMKII [5, 11], which is a kinase that typically exists as a 12-subunit, double hexamer structure that autophosphorylates neighbouring subunits to maintain those subunits in an open and active state [20]. Among many other actions, active CaMKII phosphorylates post-synaptic AMPA receptors to enhance their efficacy, which is a key expression mechanism for LTP and memory [21] and produces other synaptic alterations [22]. This concept was highly influential because it addressed both problems: (1) LTP could initially be induced by activation of existing, local CaMKII double hexamers by calcium influx through the NMDA receptor and (2) new, unphosphorylated subunits could be incorporated into CaMKII double hexamers without interrupting its activity state and, thanks to autophosphorylation of neighbouring subunits, perpetuate its activity state in a way that is immune to molecular turnover and local to the synapse at which potentiation originally occurred [23–25].

Even the subsequent discoveries that hippocampal synapses were bidirectionally modifiable, allowing low frequency (1 Hz) stimulation to induce calcium-dependent, Hebbian long-term depression (LTD) [26, 27], and that this LTD was mediated in part by protein phosphatases [28–30], was presaged by Lisman in a modified CaMKII model [12]. In this updated model, phosphatases such as calcineurin respond to lower calcium concentrations than CaMKII and act to dephosphorylate CaMKII targets and CaMKII itself, thereby setting up a bistable switch at synapses that can adopt a potentiated or a depressed state. Through its elegance and simplicity, this overall model has been highly influential, and it became the textbook example of how lasting synaptic modification may occur at individual synapses. Much experimental evidence supported the model as well, notably the demonstration that phosphorylation of the T286 residue of αCaMKII, which maintains CaMKII in an open state and is the key site for sustained activity through autophosphorylation, is required for LTP and memory [31]. However, two major experimental observations were strongly at odds with the model: First, blockade of CaMKII activity with selective inhibitors prevents LTP induction and learning, but does not prevent LTP maintenance or memory if applied very shortly after induction/learning [32–34] and, second, CaMKII activity is not sustained for more than a few minutes after LTP induction [35–37].

While CaMKII has failed to be fully anointed as the molecular basis of the engram, PKMζ, which is an atypical isoform of the calcium-dependent protein kinase (PKC), has emerged as an alternative candidate to support long-lasting, synapse-specific potentiation and memory. The model of how PKMζ may mediate synapse-specific potentiation and remain immune to molecular turnover is somewhat different from CaMKII, but no less elegant [38]. In this case, mRNA transcripts encoding PKMζ (transcribed from the PRKCζ gene), are freely available within the cell, but translation into PKMζ protein is blocked by a protein called PIN1 (protein interacting with NIMA1) [39]. Only once the LTP/learning event activates calcium-dependent synaptic signalling, which includes CaMKII, is this PIN1 block on the translation of PKMζ removed [40]. PKMζ can then act to increase the number of post-synaptic AMPA receptors through interactions with NSF-dependent AMPA receptor trafficking [41, 42]. Critically, the newly-synthesized PKMζ then suppresses further PIN1 activity, perpetuating its own synthesis [39]. For this model to work as a synapse-specific potentiation system, it is assumed that protein synthesis is localised to each synapse due to proximal positioning of ribosome complexes, for which there is substantial evidence [43, 44].

A wealth of evidence is consistent with this kinase serving as a molecular basis of the engram. Most strikingly, application of an inhibitor peptide, ZIP, which was designed to be highly selective for PKMζ, reverses long-established LTP and memory [45, 46]. Additionally, the synthesis of constitutively active PKMζ is increased for at least a month after LTP is induced [47], in contrast to observations of CaMKII activity [35, 36]. These findings have had a dramatic impact on the field of learning and memory because, until very recently, there had not been an example of a molecule that could be targeted to erase established LTP and memory. As with CaMKII, however, major experimental counter-evidence clouds the waters. First, genetic knockouts of the PRKCζ gene (encoding PKCζ and PKMζ) do not prevent LTP or learning and memory in a mouse [48, 49] and, second, the specific inhibitor ZIP is equally effective at erasing LTP and memory in these knockout mice [49, 50], demonstrating that it is not as specific as it was designed to be. Thus, both CaMKII and PKMζ are compelling at a conceptual level, but their supporters are left with some major explaining to do.

In his article ‘Criteria for Identifying the Molecular Basis of the Engram’, John Lisman has built a case for CaMKII as the strongest candidate for the molecular basis of the engram [10]. He does so by comparing it directly to PKMζ and asking how well the experimental evidence for each stack up in relation to key criteria: First, that the enzyme is necessary for LTP/memory. Second, that its overactivity will saturate synaptic strength and occlude LTP/memory and, third, that specific blockade after induction/learning will lead to LTP/memory erasure. He also discusses a fourth consideration, which is how well each mechanism could serve as a local maintenance system. While Lisman has made a compelling case, it is important to be clear that the evidence has been selectively presented. First, Lisman argues that there is more evidence for CaMKII being necessary for LTP/memory than there is for PKMζ, based on the observation that LTP and memory are retained in PKCζ knockout mice [48, 49], while the T286A αCaMKII point mutant mice, that do not maintain autonomous activity of αCaMKII through autophosphorylation, show very little LTP and highly deficient hippocampus-dependent memory [31]. However, this is a somewhat unfair comparison. Lisman neglects to discuss the fact that the full αCaMKII knockout mice, which provides the most direct comparison with the PKMζ knockout mouse, retain a substantial amount of LTP at these synapses [51, 52] and show slowed hippocampus-dependent learning that can nonetheless reach a normal asymptote over time [53], likely due to compensation by the beta isoform of CaMKII [54]. This type of genetic compensation is precisely the explanation that seems most likely for the maintained plasticity and memory in the PKCζ knockout mouse, where the iota/lamda isoform of PKC likely supports most duties in the absence of PKMζ [50]. Furthermore, even where LTP appears completely absent at the Schaffer collateral-CA1 synapses in the T286A point mutant, LTP can be induced by alternative NMDA receptor-dependent means at medial perforant path-granule cell synapses in the dentate gyrus subfield of the hippocampus [55], and lasting memory can be attained with overtraining [56]. In the strongest sense of ‘necessity’ for LTP/memory maintenance, therefore, neither CaMKII nor PKMζ meets the criterion. However, there is compelling evidence that, under normal circumstances, both play a critical role in many forms of synaptic plasticity and memory.

A more complex criterion to address is the stipulation that overactivity of a memory mechanism should saturate synaptic strength and thereby occlude LTP/memory. As presented by Lisman in ‘Criteria for Identifying the Molecular Basis of the Engram’, overactivity of CaMKII increases synaptic strength and prevents LTP induction or learning [9, 57], while a similar treatment for PKMζ enhances LTP and learning [58]. Thus, CaMKII appears to meet the occlusion criterion while PKMζ fails. However, if we were to select a different set of representative studies, then overexpression of activated CaMKII has been shown to enhance some forms of LTP [59] while overexpression of activated PKMζ increases synaptic transmission and blocks further LTP [60]. Thus, there is little consensus yet as to which of these two molecules best meets the occlusion criterion, reflecting the fact that, despite much excellent work, the true molecular basis of the engram is not fully established.

Next, Lisman argues that while ZIP is highly effective at erasing LTP and memory, his laboratory has identified better ways of doing precisely the same for CaMKII. They find in slices that a dose (20 μM) of the cell-permeable peptide tatCN21 that both inhibits CaMKII and interferes with the association of the enzyme with postsynaptic NMDA receptors is sufficient to depress basal synaptic transmission and erase established LTP [61]. Additionally, a transiently-expressing herpes simplex viral (HSV) vector, which temporarily produces a dominant negative form of CaMKII, erases established place avoidance memory [9]. Moreover, he states that an additional important test is included in these experiments that has not been conducted in the key ZIP studies [45, 46, 60], namely that synapses are re-potentiated, and animals re-learn after erasure, demonstrating that the necessary synaptic machinery remained intact despite the presence of a foreign agent. This is a crucial point, and more effort should certainly be made to evaluate the erasing effects of ZIP, given some indication that ZIP may damage cells [62] or target other neural processes than synaptic function [63]. However, it is worth noting that evidence exists that plasticity can be restored after erasure by ZIP [64] and that alternative pharmaceutical inhibitors of PKM catalytic activity also erase established LTP [60].

The findings with a high dose of the inhibitor tatCN21 stand in contrast to the effects of a lower dose. Unlike other pharmaceutical inhibitors such as KN62 and KN93, tatCN21 at a dose of 5 μM inhibits CaMKII phosphorylation of substrates even when the enzyme has been switched into an activated, autonomous state [33, 61]. Interestingly however, at this dose LTP maintenance is unaffected. This finding therefore calls into question the original model of CaMKII as a self-perpetuating ‘memorase’ [33]. To reconcile these findings, it is necessary to further postulate existence of a critical contribution of the physical association of CaMKII and NMDA receptors in the postsynaptic density. At high doses, tatCN21 and related peptides disrupt CaMKII binding to the NMDAR [61, 65], and it is argued that the dose dependency of the tatCN21 peptide on LTP maintenance is reflective of the need for a CaMKII-NMDAR complex in LTP maintenance. Sanhueza and Lisman have suggested that this interaction can contribute to the structural modifications that are believed to support long-term functional modifications of synapses [65], and more work is required to understand what these structural changes may be. While this new model revives a critical role for CaMKII in long-term synaptic modifications underlying memory, it is somewhat removed from the elegance of the original Crick/Lisman hypotheses [4, 5, 11, 19].

A last point for discussion is Lisman’s contention that it is difficult to justify PKMζ as a synapse-specific mechanism of maintenance given the fact that that it appears to be increased in expression throughout neurons after learning and has a major effect on gene expression at the nucleus as a result [66]. Bearing in mind that PKMζ can only be in an active state, this contrasts dramatically with the observation that CaMKII activity remains very local to the spine where it is induced [36, 37], and suggests that if PKMζ were the primary molecular basis of the engram it would likely exert its influence on surrounding synapses, a prospect that is clearly at odds with the input specificity of LTP [16] and Hebbian synaptic plasticity in general [13]. Lisman certainly raises a valid concern here, but it is also perfectly possible that PKMζ does not reach sufficient concentration in neighbouring spines to suppress PIN1 activity and switch the synapse into a stable ‘memory’ state. This is an issue that requires serious experimental investigation. The proposal from Lisman that PKMζ is, for this reason, better suited as a heterosynaptic scaling mechanism does, however, seem flawed, as scaling is proposed to be a homeostatic mechanism that potentiates synapses after prolonged periods of reduced neural activity [67, 68], whereas it is pronounced, event-related stimulation of neural activity that leads to PKMζ expression [47, 69, 70]. It would be very useful to image PKMζ distribution and concentration as LTP is induced at single synapses, as this would provide some insight into its differential contributions to synapse-specific and whole-cell memory mechanisms.

John Lisman made an extraordinary contribution to neuroscience. He strived to pull together threads of data from multiple sources to clearly articulate models for memory storage, lay out the critical tests of these models, and challenge the field (including his own laboratory) to refute them with rigorous experiments. His voice will be sorely missed in this debate. After much work from many scientists we cannot question the idea that CaMKII autophosphorylation is required for learning and LTP induction, proving that the ‘memorase’ property of the kinase does at least bridge a critical period between NMDA receptor opening and AMPA receptor modification. CaMKII may well play a key role in memory maintenance, although through a different mechanism than originally envisioned, and these are just two parts of a much larger scientific legacy. For science to progress there must always be innovative ideas, but there must always be rigorous testing and criticism of these ideas to determine their validity. John Lisman has been a powerful force in both processes, as illustrated in polemical pieces such as ‘Criteria for identifying the Molecular Basis of the Engram’. His article succeeds in illustrating that the evidence for CaMKII as a memory maintenance mechanism is no weaker than that for PKMζ. The clearest message that we can take from this is that much more work needs to be done to understand the molecular basis of the engram. Whether there is truly one mechanism that governs memory remains open to question. Typically, evolution would not arrive at such a fragile solution, and tends to re-purpose a multitude of existing mechanisms to fulfil similar, overlapping roles [71]. Both PKMζ and CaMKII are conserved systems, being present and operational in synaptic plasticity in both invertebrates and vertebrates [72, 73], but there are many such conserved candidate systems. It therefore seems unlikely that either CaMKII or PKMζ serves as the singular molecular basis of the engram [74], but there is clear and compelling evidence that each plays a central role.

**Commentary on ‘** ***Criteria for identifying the molecular basis of the engram (CaMKII, PKMζ)*** **’.**

*Contributed by Karl Peter Giese.*

How the brain stores memory is a fundamental question in neuroscience. John Lisman pioneered the molecular investigation of this question. Back in 1985 he hypothesized that autophosphorylation of a kinase may be a molecular switch that leads to persistent, memory-storing signaling at synapses [4; for independent pioneering work see, 19]. Over the last three decades John Lisman’s theoretical work inspired many experiments that eventually gave rise to a very sophisticated CaMKII hypothesis of memory storage [5–7, 10]. Later, a different hypothesis emerged, stating that PKMζ could provide memory-storing signaling at synapses [38]. Currently, it is unclear whether the CaMKII hypothesis and the PKMζ hypothesis are mutually exclusive, or whether both kinases together mediate memory storage (see also, [74]). In his last review John Lisman critically discusses the evidence for and against the two hypotheses [10]. He also explains fundamental concepts and introduces criteria for the molecular basis of memory storage. Whilst I think that this review is very important, I disagree with some points, as follows.

One of the proposed criteria is the saturation/occlusion test. The idea behind this test is that overexpression of an activated form of the memory-storing protein after memory formation is completed should increase most synaptic weights, preventing memory access [9]. However, this test is confounded if the protein of interest is involved in retrieval-induced memory destabilization that precedes reconsolidation/restabilization [75]. Specifically, this is the case for CaMKII. Using inducible overactivation of CaMKII, Joe Tsien’s lab showed that increased CaMKII at the time of retrieval causes memory loss [76]. Further, they established that this is not due to deficits in accessing the acquired memory, because switching off CaMKII overactivation did not restore the lost memory. Therefore, it was concluded that CaMKII overactivation enhances memory destabilization, leading to memory erasure, and that this process requires protein degradation [77]. Consistent with this idea, CaMKII regulates protein degradation at the synapse [78] and blocking CaMKII activity impairs retrieval-induced protein degradation and memory destabilization [79]. Furthermore, increasing CaMKII activity by knockdown of an endogenous CaMKII inhibitor protein leads to enhanced memory destabilization [80]. Rossetti et al. [9] found that after memory formation overexpression of constitutively active CaMKII impairs memory and in his review John Lisman writes that these results would ‘support the concept that memory is mediated by an LTP-like process dependent on CaMKII’. However, considering the evidence discussed above, the occlusion/saturation test for CaMKII is inconclusive; it is very likely that the memory impairment observed by Rossetti et al. [9] is caused by enhanced memory destabilization rather than a deficit in memory access.

An intriguing alternative to the saturation/occlusion test is an enhancement test. The idea behind such test is that overexpression of the memory-storing protein after memory formation (including consolidation) would lead to increased memory due to elevated expression at relevant synapses. Yadin Dudai’s lab demonstrated the enhancement of previously consolidated memory by PKMζ overexpression, supporting the PKMζ hypothesis [81].

In addition to a saturation/occlusion test, John Lisman proposed a necessary test as criteria. This test involves blocking the function of a candidate memory-storing protein already at the time of training. The necessary test is most powerful for exclusion of a candidate mechanism when the blockade does not prevent memory storage. Both αCaMKII and PKCζ /PKMζ knockout mouse lines can form spatial memory [50, 54]. Thus, in principle, these knockout studies reject both the αCaMKII and the PKMζ hypothesis. However, it is not known whether long-term retention of spatial memory is impaired in these knockouts. Moreover, both knockout mouse lines have a compensatory upregulation by related kinase isoforms [50, 54]. Such compensation does not occur in some knockin mutants. In calcium/calmodulin-binding-deficient- αCaMKII (T305D) mutants there is no compensatory translocation of ßCaMKII into the postsynaptic density and spatial memory formation is prevented [54]. Threonine-286 autophosphorylation-deficient αCaMKII (T286A) mutants also cannot form spatial memory, even when environmental enrichment is provided [31, 82]. Thus, αCaMKII knockin studies pass the necessary test, unlike the αCaMKII knockout studies. However, it should be pointed out that aversive, hippocampus- and amygdala-dependent memories can be formed in the T286A knockin mutants, indicating that the threonine-286 autophosphorylation of αCaMKII is not required for some types of memory storage [34, 56, 83, 84]. Therefore, the model provided by Rossetti et al. [9], and discussed in John Lisman’s review, may only apply to spatial memory storage and not storage of other types of memory.

The key test to determine the necessity of a memory-storing molecule is the erasure test. The erasure test consists of functional inactivation after memory has been formed and this inactivation should cease before memory is tested (to exclude an impact on retrieval). Many studies have shown that treatment with ZIP (PKMζ inhibitory peptide) erases established memories [85]. Almost all types of memories seem to be sensitive to ZIP erasure. However, ZIP not only blocks PKMζ, but also other molecules [48–50]. Thus, currently there is no erasure test to support the PKMζ hypothesis. In contrast Rossetti et al. [9] suggest that they have carried out a successful spatial memory erasure by blocking CaMKII function. However, in my opinion more experimental evidence is needed to assure that Rossetti et al. specifically blocked CaMKII function. They overexpressed αCaMKII with a point mutation (K42 M) that blocks its catalytic activity. Unfortunately, Rossetti et al. did not perform a biochemical characterization of this overexpression, addressing, for example a change in CaMKII activity in the postsynaptic density. Other studies with heterozygous K42 M knockin mutants (expressing 50% wild-type αCaMKII and 50% mutated αCaMKII) have not found a dominant-negative effect on CaMKII activity in forebrain [84]. Moreover, John Lisman’s lab showed that overexpression of the K42M mutant impairs synaptic transmission in a dominant-negative fashion in hippocampal slices [86]. However, K42M knockin mutants do not show impaired synaptic transmission in the hippocampus [84]. Taken together, this suggests that the K42 M overexpression might lead to unwanted side effects that might be the cause of the memory erasure.

In conclusion, John Lisman’s review presents very important conceptual points regarding the molecular basis of memory storage. It also analytically discusses the PKMζ hypothesis, but is less critical of the CaMKII hypothesis. In my view, both hypotheses could be correct, but more experimental evidence is needed to ascertain this.

**Commentary on “Criteria for identifying the molecular basis of the engram (CaMKII, PKMζ)”.**

*Contributed by Ji-il Kim, Pojeong Park and Bong-Kiun Kaang.*

John Lisman was a respected scholar with a great passion for science. Throughout his life, he made outstanding achievements in various scientific fields. In particular, he devoted most of his career to answering the question, “What is the molecular basis of memory?”

He hypothesized that a molecular switch triggered by recent activities could maintain synaptic potentiation and memory [4]. Once the molecular switch is activated, memory can be maintained through a reverberating positive feedback loop. Lisman found that Ca2+/calmodulin-dependent protein kinase II (CaMKII) has many properties that met his hypothesis and followed up on this during his lifetime to prove that this brain-enriched complex is the “memory molecule.”

In Lisman’s posthumously published paper, we can appreciate his unique and great ideas regarding his original question, “What is the molecular basis of memory?” [10]. In particular, unlike the general perspective of the engram studied at the circuit, cell, and synapse levels [87], he stated that the engram constitutes the molecular changes by which a memory is stored in the brain. In addition, he marked a milestone in the analysis of the molecular engram by presenting appropriate criteria in his paper [10].

In the first part of this paper, Lisman described the relationship between memory and long-term potentiation (LTP) at a glance and discussed why and how studying LTP contributes to understanding the nature of memory. In a similar vein, he also emphasized the importance of understanding the molecular mechanism of LTP maintenance to identify the memory molecule.

In addition, Lisman suggested three types of tests (Necessary, Saturation/Occlusion, and Erasure tests), which would be used to evaluate the role of molecules in the maintenance of LTP and memory. By comparing two major candidates of the memory molecule, protein kinase M ζ (PKMζ) and CaMKII, based on his three types of tests, he suggested that CaMKII, rather than PKMζ, is more appropriate as the memory molecule.

Subsequently, Lisman divided the LTP process into three different phases: Induction (the triggering of signal cascades by, for example, calcium), Maintenance (the throwing of a molecular switch such as autophosphorylation of CaMKII) and Expression (involving the downstream targets (AMPA receptors) of the activated Maintenance molecule(s)). Memory, Lisman argues, will only be permanently erased by manoeuvres that attack the molecular mechanisms responsible for Maintenance. This subdivision is of importance to understand whether a candidate molecule is specifically responsible for LTP maintenance, rather than for other phases. Therefore, Lisman emphasized that before a candidate molecule is interpreted as being important in the maintenance process, it should be confirmed whether the impairment of LTP is caused by a perturbation during the induction or expression processes. Data interpretation based on these subprocesses might reduce any confusion in searching for the memory molecule regulating the maintenance process, which underlies storage of the engram. In summary, Lisman’s insightful theories, such as of the three types of tests for evaluating candidate molecules and interpretation based on subprocesses, will serve as significant milestones for future memory research.

Despite Lisman’s significant contributions, some important questions remain that future generations should pursue to fully understand the molecular basis of memory.

1. It is clear that synaptic potentiation occurs after appropriate stimuli, and the synaptic potentiation between engram cells underlies memory storage [88]. Considering the relationship between synaptic potentiation and memory, Lisman insisted that maintenance of synaptic potentiation by the molecular switch (that is, CaMKII) is the mechanism that maintains memory over a long period of time. In other words, the enhancement of the synapses in which the potentiation occurs during learning should be maintained persistently to store the memory.

However, the place where memory is stored in our brain might be highly dynamic. It is well known that episodic memories in rodents are initially stored in the hippocampus for several weeks, but are gradually transferred to cortical areas [89]. Moreover, recent studies have demonstrated that engram cells in various brain regions are continuously changing [90–93]. According to these results, synapses that store memory are seemingly not a static, but a highly dynamic structure after learning. If the engram cells are dynamically changing, then the memory-storing engram synapses might also be dynamically changing, and the enhancement of synapses that are initially activated during learning may not need to be sustained for a long time to maintain memories. Therefore, although it is necessary to study how molecular cascades could be maintained over a long period of time at the synapse level, future studies will need to adopt a systems approach to explain how a qualitatively similar memory can be preserved in the light of memory engram dynamics.

2. In addition to CaMKII, many other molecules such as PKMζ and cytoplasmic polyadenylation element-binding protein (CPEB) are known to be involved in memory maintenance [38, 94]. As Lisman insisted, CaMKII might be the only molecule that meets the three criteria, but there are considerable data that other molecules are also involved in memory maintenance. In particular, PKMζ is of interest as a key molecule in memory maintenance because it could be constitutively active with the lack of a regulatory subunit [38, 50]. Some evidence suggests that PKMζ is crucial to maintain synaptic potentiation not only in memory, but also in chronic pain [95] and drug addiction [96]. In addition, PKMζ is also involved in epigenetic mechanisms, which might be related to long-term regulation of gene expression for memory maintenance [66]. Therefore, it is necessary to reconcile these data with an integrated view.

Interestingly, memory can also be abolished when a constitutively active form of CaMKII is overexpressed [9], whereas an enhanced memory has been observed following PKMζ overexpression [81]. Considering the role of CaMKII, which is also important in memory induction, this memory deficit could be a result of indiscriminate spine maturation by excessive CaMKII activity, which might destroy patterns that encode the memory [9]. In contrast, PKMζ, which is known to be insufficient to induce spine maturation, may be activated by another mechanism (such as phosphoinositide-dependent protein kinase-1) to maintain memory [97]. Thus, PKMζ might not be able to cause indiscriminate spine maturation just by its overexpression, but it could possibly induce over-maturation by functioning to a greater extent in engram spines where maturation has already occurred. Therefore, the molecular cascade including CaMKII and PKMζ should be further investigated to better understand the detailed molecular mechanisms of memory maintenance.

John Lisman’s contributions to science, and especially his contributions to the molecular basis of memory were original, wide-ranging and important. Future generations must answer the remaining questions to better understand the biological foundations of memory.

**Commentary on “Criteria for identifying the molecular basis of the engram (CaMKII, PKMζ)”.**

*Contributed by Mary B. Kennedy.*

John Lisman was a devoted and serious neuroscientist who pursued his chosen problems and ideas with passion. Among these was a question which he formulated as: what mechanisms underlie the formation of “the engram?” The engram is an abstraction that means slightly different things to different people. Some consider it to be the more or less permanent neural circuits that underlie specific episodic memories. To John, it was “the molecular changes by which a memory is stored in the brain.” Of course, one can easily assume that the molecular changes he refers to are those that form and maintain the more or less permanent neural circuits that underlie specific episodic memories.

My own career has been devoted to understanding what I refer to as the biochemical mechanisms that regulate synaptic strength in excitatory synapses and thus allow synapses to adapt their signaling patterns to environmental input, and to store memories. The slight differences in emphasis between my conception of synaptic regulation and John’s statements about “the engram” go a long way toward explaining why he and I so often disagreed profoundly about the interpretation of various experiments and about the experimental way forward to understand synaptic regulation and memory.

When I was an assistant professor, I and my students set out to use the protein chemistry methods that I still revere, to discover and understand the function of regulatory proteins in the postsynaptic density. My first students and I began by purifying a calmodulin-dependent protein kinase (later named CaMKII) that, as a postdoc in the Greengard laboratory, I had first measured in brain homogenates and identified as located in a particular fraction of proteins eluted from a DEAE-cellulose column. We quickly realized that it was highly abundant in brain, and that it appeared to be a quantitatively significant component of the postsynaptic density [98–100]. While studying the enzymatic properties of the purified protein, my student, Steve Miller, noticed that the kinase activity seemed to retain some phosphorylating activity in the absence of calcium and calmodulin after it had phosphorylated itself in the presence of these two activators. During a visit to my lab, John pointed out that this behavior might fit a theoretical model he had proposed regarding a way for an activated state to outlast turnover of individual molecules and we mentioned this in our paper [4, 20].

The notion of a calcium-dependent switch excited many neuroscientists and John began assuring me that we might have discovered “the memory molecule.” This notion, and the reaction of some neuroscientists to it, began to disturb me. My conception of regulatory biochemistry did not and does not include the notion that a single molecule would constitute “memory.” I had, and have, great respect for the complexity of molecular evolution. I believe that subcellular “states,” especially powerful synaptic connections that are important for brain function, must be generated and maintained by intricately controlled and robust mechanisms. Even the pathways of bacterial metabolism are filled with feedback regulation and alternative pathways. How much more complexity must have evolved to accurately regulate a process as subtle and important as mammalian episodic memory and insure its robustness? I resisted the idea that CaMKII was “the memory molecule” for this reason. There is no “memory molecule.” Synapses are regulated by the mutual interactions of a whole network of proteins. It was clear then and is even clearer now, that CaMKII is a critical component of synaptic regulation because it is abundant in the brain and postsynaptic density, it responds to calcium coming through NMDA receptors, and retains its activation for a short period that is delimited by the opposing activity of protein phosphatases. We now know that CaMKII feeds back to AMPA and NMDA receptors by phosphorylating them, and it can initiate changes in many downstream regulatory enzymes including nitric oxide synthase, synGAP, and regulators of the actin cytoskeleton. But it is also true that calcium and calmodulin in the spine directly regulate a large number of other processes, including several that depend on cAMP-dependent protein kinases, and various forms of protein kinase C. Thus, I have always believed that an important way forward to understand synaptic regulation is to study in vitro the mutual interactions among the regulatory molecules in the postsynaptic density and spine, test whether and when those interactions are important for synaptic function, and learn the quantitative kinetic parameters that govern these interactions so that we can gradually account completely for observed functional changes by modeling the dynamic action of biochemical regulatory loops and pathways (for a beginning effort see [101]).

John took a very different approach. His earliest training was in physics, and his primary technical expertise was in electrophysiology. He worked by using logic to create abstractions like “the engram”, or “induction, expression, and maintenance;” then trying to devise ways that he and others could use pharmacology, electrophysiology and sometimes genetics to deduce molecular mechanisms. To my mind, these abstractions involve myriad assumptions that are often unfounded and tend to obscure the subtleties of actual molecular mechanisms. Each of these “phases” of LTP that can be measured with an electrode encompasses many underlying molecular mechanisms. I think that a defining disagreement between John and me, and indeed between me and a host of synaptic electrophysiologists, is that one cannot tease out and understand the molecular mechanisms of synaptic plasticity with electrodes, pharmacology, and genetics alone. Each of these methods is useful, but without an understanding of the subtleties of biochemical interactions of proteins in a regulatory network, the picture will always be incomplete, and may often be badly oversimplified. John was surely not alone in his conviction that he could reason through fundamental molecular mechanisms of learning with logic and electrophysiological experiments in intact tissues and animals. This thinking still permeates the NIH review panels at every level and has damaged funding of in vitro biochemical studies. In turn, this situation has damaged the ability of basic neuroscience to provide the knowledge that pharmaceutical companies need to identify possible therapeutics for Alzheimer’s disease, mental illnesses, and autism spectrum disorders.

It is evident that both CaMKII and PKMζ play crucial roles in the mechanisms of synaptic plasticity and in maintaining synaptic strength. There is no reason to see their importance as mutually exclusive. A more useful way forward will be to search out the crucial “substrate proteins” that these two fascinating enzymes regulate. It is even possible that CaMKII plays an important structural role in the postsynapse. In vitro biochemical work will be needed to fully understand the interlocking functions of the synaptic regulatory networks.

I am inclined to agree with a comment by Richard Morris [102] in a discussion of a recent paper from the Sacktor lab on PKMζ [50]. Morris reminds us that a “molecule implicated in memory retention really does (not) need to be sustained throughout the lifetime of a memory. An alternative possibility is that it may trigger structural changes that are, in turn, mediated by other molecules (such as actin): thus, with this job done, our memory molecule can gracefully depart the scene to play upon another stage. Such structural changes could then be faithfully recycled during routine protein turnover, with these proteins being unaware, so to speak, that they are sustaining a memory.” When a synapse is made more powerful by the addition of more AMPA receptors, more active zones, a larger spine and postsynaptic density, etc., the collective energy embodied in the myriad protein affinities imparts great structural stability. Removal or dislodging of any one or two or three proteins will not disrupt the structural stability imparted by all the other associations. Thus, normal cellular repair and maintenance processes could continue to maintain the larger synapse once it is established. These cellular processes are not perfect; yet we all know that memory isn’t perfect either and memories are often lost or altered as we age.

John Lisman made many contributions to neuroscience. His passion and his provocative comments and ideas will be missed.

**The non-erasable memory of John Lisman.**

*Contributed by Richard G M Morris.*

John Lisman was a man who was infectiously curious, read voraciously, thought deeply and was genuinely interested in what others had to say. He was a listener. He was both passionate about science and generous to others around him who were pursuing their scientific interests; indeed the recent appreciations of him by friends and colleagues [103] are very moving. He was a man with diverse scientific interests ranging from phototransduction, the possibility that autophosphorylation of CaMKII could be a molecular switch for memory, neural oscillations and working memory storage and, later in his career, the puzzle of schizophrenia. This diversity led him into contact with all manner of different people in neuroscience that don’t often connect. John Lisman was a man who loved science and for whom science was - beyond family - his life.

In his Paths to Discovery contribution to the 3rd edition of the Bear, Connors and Paradiso textbook of neuroscience [104], John describes how he came up with the idea of an autophophorylation as a molecular switch for memory. Inevitably, it was during a walk along a beach - the best ideas often come near the sea:

*“My eureka moment …. was that a group of autophosphorylating kinase molecules localized at a synapse could make a stable switch. During LTP induction, these molecules would become phosphorylated, and this would make them active. If a kinase molecule was dephosphorylated or replaced in the course of protein turnover, it could be phosphorylated by other members of the group. The switch could stay on, perhaps indefinitely, and this showed how unstable molecules could produce stable information storage.”*

In this final paper on which we are commenting [10], John asserts that the definitive evidence that CaMKII autophosphorylation could be a mechanism of memory comes not from correlational data showing that this process occurs in a lasting manner in response to neural activity that may be associated with memory formation in synaptic or behavioural models - for such data (important as they are) are merely suggestive of a memory storage mechanism. Rather, it is from a particular type of experiment from which a causal inference can be drawn. This is his “erasure” test. The argument is attractively simple: if CaMKII autophosphorylation is a mechanism of memory storage, memory must disappear if you turn it off.

The argument has depth too for, in Figure 1, he defines the critical distinction between an induction process, a maintenance process and an expression process with respect to the making of a lasting memory trace, and at several points in his article asserts (correctly in my view) that evidence from expression experiments is not necessarily relevant to induction or maintenance processes. We must keep these distinctions separate. However, while not in any way demurring from this argument, I am not convinced it is sufficient - for a more ‘top-down’ perspective on memory would distinguish the dissociable processes of memory-encoding, storage, consolidation and retrieval. Re-activation and retrieval did not seem to be on John’s radar and this omission from his thinking may be critical. From a psychological perspective, the supposition is that memory traces in long-term memory can be active or can become dormant (i.e. ‘inactive’) over weeks, months or even years. And then - magically as it were - be reactivated and retrieved. Does the autophosphorylation idea require that this biochemical process is going on discreetly at dormant synapse after dormant synapse across these time-periods? Here I have a certain suspension of disbelief for a pre- and post-synaptic structural solution at the synapse feels (to me) much more economical. A metaphor may be helpful here to get across the general idea: Consider the task of a supermarket store-manager trying to maximise his or her sales. They need to devote sufficient length and space in an aisle to the sale of different items, and not allow an item that doesn’t sell very well to occupy too much space. Given this - the range of (say) coffee items might take up a metre or two, but should not occupy more. The memory in the system here is the length of the shelf devoted to coffee, itself a reflection of manager’s experience of how quickly they are taken off the shelves by the shop’s customers. But the items - the coffee jars - are temporary and unstable. In this way, stability and instability co-habit happily, even though there is an analogy to the protein turnover that rightly concerned John in his thinking about CaMKII. His ideas about “slots” in the PSD was very much in the same spirit.

But this need not mean Lisman’s autophosphorylation idea is wrong. To the contrary, if we build into his framework the important distinction between cellular (i.e. immediate) and systems consolidation (longer-term), it seems entirely possible that sustained CaMKII autophosphorylation during the initial cellular phase may be critical for memory as he thought. What is important about this division into two qualititatively distinct consolidation systems, from the perspective of evolution, is that a cellular consolidation process takes things ‘off-line’ from the perspective of cell firing (the relevant brain cells can get on with other new learning). However, this biochemical process serves, for a while, as an on-line ‘long-list’ of what is destined to be retained indefinitely in the brain’s lasting long-term memory systems. That is, post-processing, a body of information is being retained for potentially quite long periods (hours, certainly; days, maybe) even though not all of it may be retained by the longer-term, and probably structural, systems consolidation process.

The data presented in Figure 4 and 5 show Lisman’s favoured ‘saturation’ and ‘erasure’ tests for establishing a role for CaMKII autophosphorylation in memory storage. In the former test, animals learn to avoid a specific place in Andre Fenton’s ingenious place avoidance task (see path data for Trial 12). At that point, an HSV vector expressing activated CaMKII is infused into the hippocampus with the expected mechanistic consequence being a disruption of the spatial pattern of synaptic weights. Memory is then poorer (but not in the control condition) on Trial 13 (6 days later). This is more of a memory retrieval test than any other, and I note that the inspiration for this experiment was Vegard Brun’s similar study in which LTP itself was saturated [105]. The ‘erasure’ test is different. In this case again, a memory is first acquired - either in a model system such as LTP or in behaviour. The idea then is to erase this memory using TatCN21 or HSV-K42 M which interferes with the ability of CaMKII to bind effectively with the NMDA receptor. When given after 4 days of training, loss of memory was demonstrated in a test conducted 10 days later after, apparently, the dominant negative construct was no longer present. This means that the loss of memory is not a transient consequence of it not being expressed well in the presence of the interfering agent. The memory trace is really gone. But the encoding system is not damaged and re-learning can occur. By any standards, this is a powerful experimental design from which strong inferences can indeed be drawn.

But where does this leave us? I share John’s assertion that “the molecular basis of memory storage is one of the most fundamental questions in cellular neuroscience”. (page 9). I am less sure that we are yet in a situation where we can all go home and say “problem solved”. Part of my reticence is that I feel uncertain about any theory that, implicitly or explicitly, implies that a specific molecule is the be-all and end-all of everything. Even in the case of DNA, which comes pretty close to meeting that sense of “problem solved” with respect to the issue of replication, there were still all manner of issues that have sensibly occupied molecular biologists for years since. With respect to neuroscience, I am not sure we are yet in a position to think that the autophosphorylation of CaMKII is quite in the DNA league, but also doubt that we can think of the problem of memory in merely a cellular way. I have already noted what feels to me to be the weakness of a theory that apparently requires a biochemical autophosphorylation process to be continuing indefinitely.

Beyond these comments, there are larger issues to do with why some memories are remembered and not others. My own reflections on this issue relate to synaptic-tagging-and-capture [106–108] and to how an initial cellular consolidation process that provides the first level of information “selection” interfaces with a systems consolidation process that serves to update existing knowledge structures such as schemas [109, 110]. One joy of my career was discussing these matters with John Lisman by email, SKYPE and in person, especially in relation to his theory with Tony Grace about the impact that novelty might have on selective retention within the hippocampus mediated via the neuromodulatory transmitter dopamine [111]. My laboratory was inspired by this idea and conducted a number of experiments on the issue, some of which went on to influence John’s own thinking. More recently, our suggestion that he, and Julie Frey earlier were right about dopamine but might not have been right about the source of the dopamine, was as intriguing to him as to ourselves. He made a point of attending our poster presentations at the Society for Neuroscience (2015) showing behavioural, electrophysiological and optogenetic data pointing to the locus coeruleus rather than the ventral tegmental area as possible cells of origin. These data were eventually published a year later [112], followed shortly by data from Eric Kandel’s lab [113] indicating the presence of dopamine release from activated LC axons. The jury is still out on this issue, but the suggestion is at least out there. Adrian Duszkiewicz and Tomonori Takeuchi both got a buzz from having John go through their posters so constructively with them - and I listened with interest. To the end, John cared about young scientists. Let us never erase our memory of such a generous, graceful and gifted scientist.
